# Management of PDA device closure complicated by severe hemolysis by transcatheter retrieval and deployment of new device

**DOI:** 10.1186/s43044-023-00343-8

**Published:** 2023-03-10

**Authors:** Aamir Rashid, Ajaz Lone, Hilal Rather, Imran Hafeez

**Affiliations:** Department of Cardiology, SKIMS, Soura, Srinagar, J& K India

**Keywords:** Hemolysis, Catheter intervention, Amplatzer duct occluder

## Abstract

**Background:**

Hemolysis after Patent ductus arteriosus (PDA) device closure is rare. Although in most cases, hemolysis settles on its own; however, in some cases it may not settle spontaneously and may require additional procedures like putting additional coils, gel foam or thrombin instillation, balloon occlusion, or removing it surgically. We report a case of adult PDA device closure who persisted with hemolysis and was managed by transcatheter retrieval.

**Case presentation:**

A 52-year-old gentleman presented to us with a diagnosis of large PDA with operable hemodynamics. Descending thoracic aortic Angio showed a large 11 mm PDA. Transcatheter device closure was done in the same sitting with a 16 × 14 Amplatzer Ductal Occluder I(ADO) device,;however, after device release, the aortic end of the device was not fully formed and there was residual flow. The next morning patient started with gross hematuria with persistent residual flow. We tried to manage with conservative means including hydration, and blood transfusion; however, residual flow persisted for 10 days and his hemoglobin dropped from 13 gm/dl preprocedural to 7 gm/dl, creatinine increased from 0.5 mg/dl to 1.9 mg/dl, bilirubin increased to 3.5 mg/dl & urine showed hemoglobinuria. As the patient continued to deteriorate it was planned to retrieve the device by transcatheter approach. 10 French amplatzer sheath was parked in the pulmonary artery near the ductus. We tried with a combination of multiple catheters and Gooseneck snare (10 mm) and finally, we successfully retrieved with a combination of Multipurpose (MP) catheter and 10 mm Gooseneck snare. After that, we closed the defect successfully with a double disk device (muscular Ventricular septal defect 14 mm Amplatzer). The patient’s hematuria settled and was discharged after 2 days with normal hemoglobin and creatinine.

**Conclusions:**

Patent ductus arteriosus ADO 1 device should not be released if the aortic end of the disk is not fully formed Patient should be carefully monitored for hemolysis if evidence of residual shunt and given supportive treatment. If conservative treatment fails, residual flow needs to be eliminated. Transcatheter retrieval although technically challenging is a feasible treatment. A muscular VSD device is a good alternative to the usual PDA device to close PDA, especially in adults.

## Background

Although transcatheter defect closure is the mainstay for treating shunt lesions like PDA, and Atrial septal defect (ASD) this method might cause hemolysis due to residual shunt or mechanical exposure of the device to blood flow [1]. Hemolysis after PDA device closure is usually associated with the presence of residual duct flow [2]. Although in most cases, hemolysis settles on its own and requires supportive care only [3, 4]; however, in some cases it may not settle spontaneously and may require additional procedures like putting additional coils, gel foam or thrombin instillation, balloon occlusion, or removing it surgically. We report a case of adult PDA device closure who persisted with hemolysis and was managed by transcatheter retrieval.

### Case presentation

**Fig. 1:**
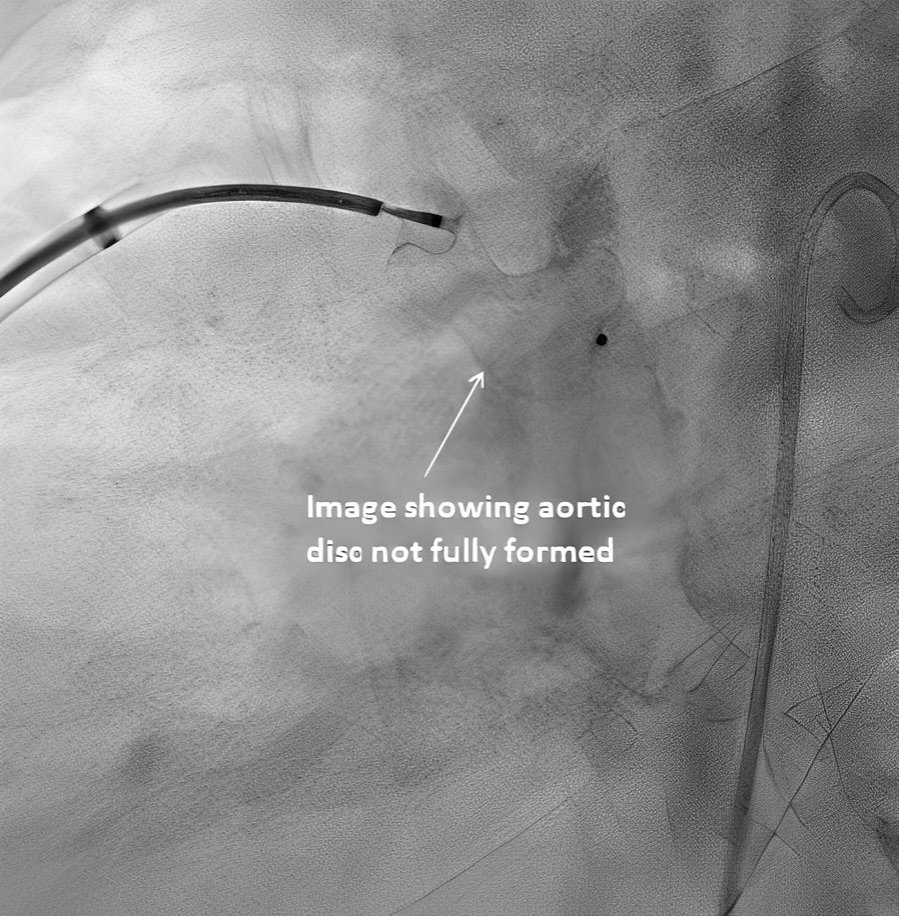
Lateral View on fluoroscopy showing aortic disk of 16 × 14 Amplatzer Duct occluder I not properly formed

**Fig. 2:**
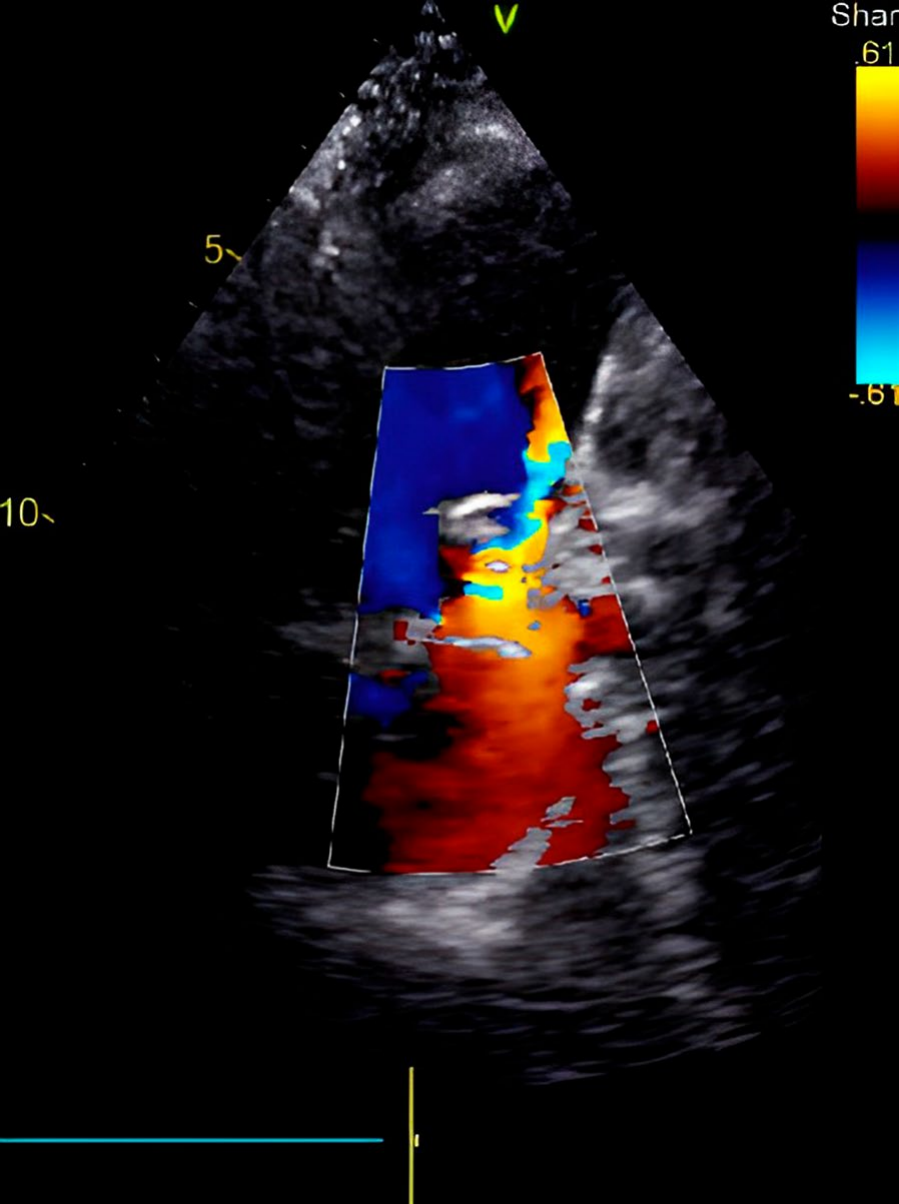
Parasternal short axis view on transthoracic echo showing significant Peri device leak

**Fig. 3:**
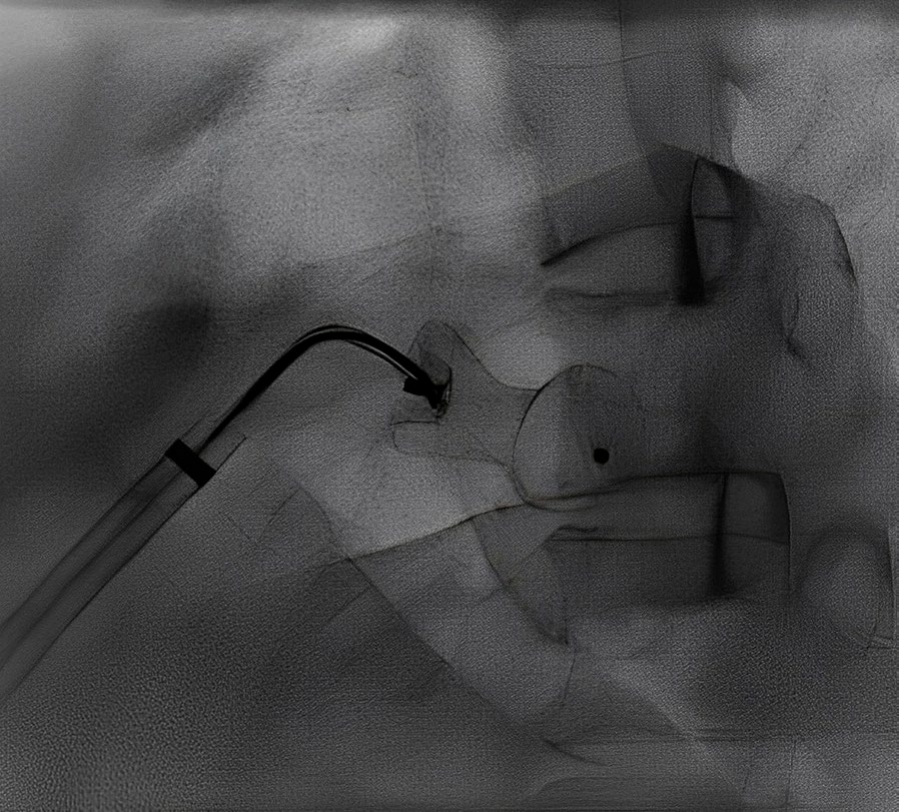
Left anterior oblique (LAO) view on fluoroscopy showing 10 mm Gooseneck snare catching screw of PDA device

**Fig. 4:**
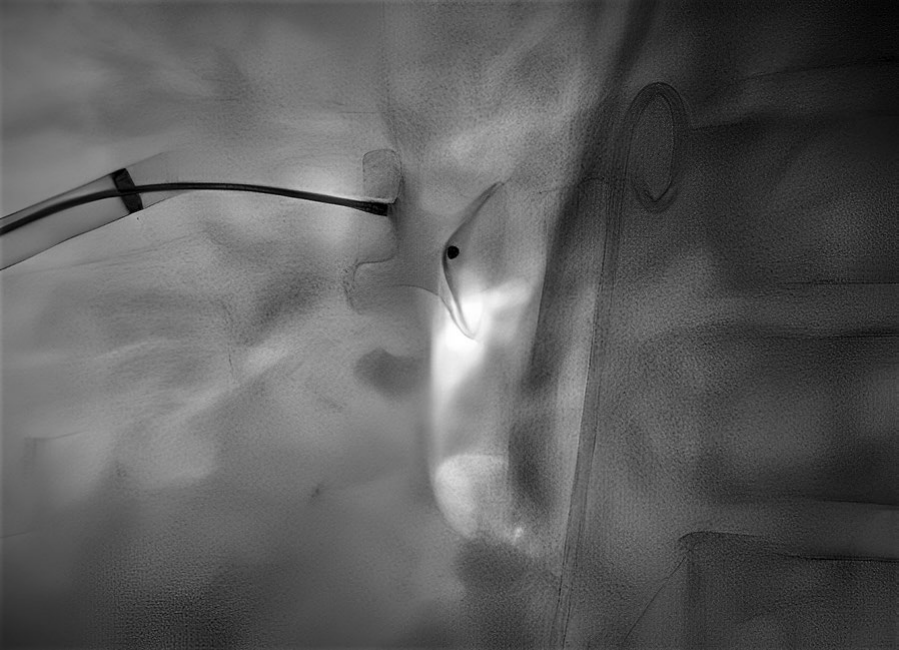
Lateral view on fluoroscopy showing 14 mm Double disk muscular VSD device deployed

A 52-year-old gentleman presented to us with New York heart association (NYHA) functional class II dyspnea on exertion (DOE). On physical examination, he was found to have wide pulse pressure, cardiomegaly, left Ventricular third heart sound (LVS3), loud pulmonary heart sound (P2), and continuous murmur in the pulmonary area. Electrocardiography (ECG) and chest X-ray (CXR) showed cardiomegaly and features of increased pulmonary blood flow. Echocardiography (Echo) showed 11 mm large PDA with dilated Left atrium & left ventricle. The right ventricular systolic pressure (RVSP) was 60 plus the right atrial mean. Cardiac cath showed operable hemodynamics with moderate pulmonary arterial hypertension{(Saturation (Superior vena cava 70%, Inferior vena cava 72%, Pulmonary artery (PA) 94%, Ascending aorta and femoral artery (FA) 100%) &Pressure (PA = 55/30 m42, FA = 210/60)}. The calculated Pulmonary vascular resistance (PVR) was 3.5 woods units and Systemic Vascular Resistance (SVR) was 14.85 woods units. Calculated Pulmonary blood flow /systemic blood flow ratio (Qp/Qs) was > 2.1. Descending thoracic aorta (DTA) Angio showed a large 11 mm PDA. Transcatheter device closure was done in the same sitting with a 16 × 14 Amplatzer Ductal Occluder I (ADO)device. After device deployment, though the device was stable the aortic end of the device was not fully formed (Fig. 1) and there was residual ductal flow. Multiple attempts were done to retrieve the device back into the sheath but each time we redeployed it, it took the same shape. Since the device looked stable it was decided to release the device, assuming that once released, the tug of the cable would go and the device will assume the proper shape and residual flow would come down. We also did not consider using another device before releasing as we thought 14 × 16 size to be appropriate since the PDA measured 11 mm at the Pulmonary end. Downsizing would have carried the risk of embolization while increasing the size may not have helped as the device was stable despite giving a good tug to the device. Although the device did not form, we assumed that it will get formed properly once we release it; however, it did not happen and after release, the aortic end of the device was still not fully formed. The residual flow also persisted. The next morning patient started with gross hematuria. Examination showed continuous murmur. Echo (Fig. 2) revealed significant residual ductal flow. Initially, we tried to manage with conservative means including hydration, and blood transfusion; however, the residual flow persisted for 10 days and his hemoglobin dropped from 13 gm/dl preprocedural to 7 gm/dl after 10 days, and creatinine increased from 0.5 mg/dl to 1.9 mg/dl, bilirubin increased to 3.5 mg/dl & urine showed hemoglobinuria. We discussed the case with our surgical team. As the patient had severe anemia with renal failure and the surgeons had to go on cardiopulmonary bypass, it was considered high risk, so it was planned to attempt transcatheter retrieval of the device failing which, the patient would be taken for surgery. After informed consent, the patient was again taken for procedure and 10 French amplatzer sheath was parked in the pulmonary artery near the ductus. Initially, we tried to screw with the device cable, however, it failed. Then combination of multiple catheters and a Gooseneck snare (10 mm) was used and finally, we successfully retrieved with a combination of a Multipurpose (MP) catheter and 10 mm Gooseneck snare (Fig. 3). After that, we closed the defect successfully with a double disk device (muscular Ventricular septal device (VSD) 14 mm Amplatzer) (Fig. 4). The patient’s hematuria settled and was discharged after 2 days with normal hemoglobin and creatinine.

## Discussion

We reported an unusual case of severe hemolysis following a PDA device closure with Amplatzer ADO I device which was managed successfully by transcatheter retrieval. The reported incidence of hemolysis is 0.5 to 3% following coil occlusion of PDA [5]. However, significant hemolysis after occlusion with Amplatzer-type duct occluders is extremely rare [3, 4]. A possible cause of hemolysis in our case was that the aortic disk was not fully formed which could have led to the failure of polyester fabrics in nitinol mesh to thrombose. There are limited case reports on the management of hemolysis post ADO I device. Godart et al. [6] reported the first case of hemolysis after implantation of 8 × 6 ADO I in an 11-month-old infant. They managed to abolish hemolysis by temporary occlusion of the aortic ampulla with a 5 Fr Berman balloon catheter. However, in our case, we did not consider it as our patient was an adult patient with large size of DTA, and balloon occlusion would not have helped. Joseph et al. [7] deployed 0.018 coils into the nitinol wire cage within the device to abolish hemolysis. We assumed the leak in our case to be both peri device and through the device. The device had not formed properly which could have led to the failure of polyester fabrics in nitinol mesh to thrombose. So, implanting an additional device/coil might have stopped the peri device but not the intradevice leak. Also implanting another device/coils involved manipulation and could have led to the embolization of the first device. Besides putting additional micro coils require additional hardware and had financial implications. We work in resource-limited settings where patients usually have limited finances. Anil et al. [8] reported the use of thrombin instillation into the ductal ampulla after balloon occlusion of the ampulla which resulted in successful flow elimination. However, in our case because of the large size of PDA and aorta, it was difficult to occlude the ampulla and there was a risk of spillover of thrombin into the systemic circulation. We instead planned to retrieve the device. Although it was technically challenging and took us fluoroscopic time of 1 h, finally we were able to retrieve it with a 10 mm snare. After that, we used a double device (Muscular VSD) to occlude the ductus. The rationale was that the double disk device has additional layers which would result in less residual flow. The device was well formed and abolished the residual flow without causing any obstruction to LPA or DTA. To the author's best knowledge this is the first reported case of management of hemolysis by transcatheter retrieval and deployment of a new device.

## Conclusions

PDA ADO 1 device should not be released if the aortic end of the disk is not fully formed Patient should be carefully monitored for hemolysis if evidence of residual shunt and given supportive treatment with IV fluids and blood transfusion. If conservative treatment fails, residual flow needs to be eliminated. Transcatheter retrieval although technically challenging is a feasible treatment and can avoid surgical procedure. A muscular VSD device is a good alternative to the usual PDA device to close PDA, especially in adults.

## Data Availability

All data generated or analyzed during this study are included in this published article.

## Abbreviations

PDA: Patent ductus arteriosus
ASD: Atrial septal defect
VSD: Ventricular septal defect
ADO: Amplatzer duct occluder
DTA: Descending thoracic aorta
DOE: Dyspnea on exertion
NYHA: New York heart association
MPA: Main pulmonary artery
LPA: Left pulmonary artery

## References

[1] Jang GY, Son CS, Lee JW, Lee JY, Kim SJ (2007). Complications after transcatheter closure of patent ductus arteriosus. J Korean Med Sci.

[2] Jung JW (2010). Recent strategies and outcomes of transcatheter closure for patent ductus arteriosus. Kor Circ J.

[3] Amoozgar H, Soltani R, Edraki M (2019). Hemolysis and its outcome following percutaneous closure of cardiac defects among children and adolescents: a prospective study. Ital J Pediatr.

[4] Khan A, Ullah Z, Ilyas S (2022). The outcome of trans-catheter closure of patent ductus arteriosus: a single-center experience. Cureus.

[5] Henry G, Danilowicz D, Verma R (1996). Severe hemolysis following partial coil-occlusion of patent ductus arteriosus. Cathet Cardiovasc Diagn.

[6] Godart F, Rodes J, Rey C (2000). Severe hemolysis after transcatheter closure of a patent arterial duct with the new Amplatzer duct occluder. Cardiol Young.

[7] George J, Asishkumar M, Zacharias TU (2002). Severe intravascular hemolysis after transcatheter closure of a large patent ductus arteriosus using the amplatzer duct occluder successful resolution by intradevice coil deployment. Catheterizat Cardiovasc Interv.

[8] Anil SR, Sivakumar K, Philip AK (2003). Clinical course and management strategies for hemolysis after transcatheter closure of patent arterial ducts. Catheterizat Cardiov Interv.

